# Protective effects and functional mechanisms of *Lactobacillus gasseri* SBT2055 against oxidative stress

**DOI:** 10.1371/journal.pone.0177106

**Published:** 2017-05-11

**Authors:** Eiji Kobatake, Hisako Nakagawa, Takahiro Seki, Tadaaki Miyazaki

**Affiliations:** 1 Milk Science Research Institute, Megmilk Snow Brand Co., Ltd., Saitama, Japan; 2 Department of Probiotics Immunology, Institute for Genetic Medicine, Hokkaido University, Sapporo, Japan; Duke University School of Medicine, UNITED STATES

## Abstract

*Lactobacillus gasseri* SBT2055 (LG2055) is one of the probiotic lactic acid bacteria. Recently, we demonstrated that feeding with LG2055 extended the lifespan of *Caenorhabditis elegans* and that the prolongevity effect was dependent upon the regulation of oxidative stress response. In this study, we assessed whether LG2055 regulated the oxidative stress response of mammalian cells. In NIH-3T3 cells and primary mouse embryonic fibroblast cells, low cell proliferation rates and high reactive oxygen species levels were observed following paraquat treatment. LG2055 treatment suppressed these responses in paraquat-treated cells, indicating that LG2055 protected against oxidative stress in mammalian cells. The mRNA expression of oxidative stress-related genes, total nuclear factor-erythroid-2-related factor 2 (Nrf2) protein levels, and the nuclear translocation of Nrf2 were increased by LG2055 treatment. These results suggested that the Nrf2-antioxidant response element (ARE) signaling pathway was activated by LG2055. Furthermore, c-Jun NH_2_-terminal kinase (JNK) was activated by LG2055 treatment and the inhibition of JNK suppressed the activation of the Nrf2-ARE signaling pathway in LG2055-treated cells. Together, these findings suggest that LG2055 activated the Nrf2-ARE signaling pathway by JNK activation, thus strengthening the defense system against oxidative stress in mammalian cells.

## Introduction

The increase of oxidative stress is harmful for an organism and is related to various diseases such as Parkinson’s disease [1], Alzheimer’s disease [2], cancer [3], heart failure [4], and atherosclerosis [5, 6]. Oxidative stress is caused by excessive reactive oxygen species (ROS), which can be produced from endogenous or exogenous sources. During cellular response to oxidative stress, antioxidant enzymes or phase II metabolizing enzymes such as superoxide dismutase (SOD), catalase, heme oxygenase-1 (HO-1), and glutathione-*S*-transferases (GSTs) are induced to protect against the oxidative stress [7]. The genes for these enzymes contain antioxidant response elements (AREs) in their promoter regions. In addition, nuclear factor-erythroid-2-related factor 2 (Nrf2), a member of the NF-E2 family of basic leucine zipper transcription factors, regulates their gene expression by binding to ARE to activate the Nrf2-ARE signaling pathway [8], which is considered to play an important role in the stress defense system against oxidative stress.

It is also considered that the oxidative stress is one of the causes of senescence [9, 10]. Normally, antioxidant defense system eliminates ROS and living organism is protected from oxidative stress. Therefore, weakening of antioxidant defense system caused by several factors, such as aging, leads to excess oxidative stress and senescence. The Nrf2-ARE signaling pathway is shown to be critical for the regulation of oxidative stress-induced senescence [11].

Lactic acid bacteria have been used in many fermented foods around the world and are therefore well adapted to human life. Some strains of lactic acid bacteria are used as probiotics, which are defined as live microorganisms that, when administered in adequate amounts, confer health benefits on the host. Recently, many beneficial effects of lactic acid bacteria such as immunoregulatory [12, 13], anti-inflammatory [14], and anti-oxidative [15] effects have been reported; thus, overall, lactic acid bacteria are considered to represent safe and valuable functional food ingredients.

In particular, we have previously studied the functions of *Lactobacillus gasseri* SBT2055 (LG2055), a probiotic lactic acid bacterium that originates from the human intestine. The beneficial effects of LG2055, such as improvement of the intestinal environment, preventive effects against influenza A virus [16], augmentation of IgA production [17], and prevention of abdominal adiposity [18, 19], have been demonstrated. Our recent study showed that feeding with LG2055 extended the lifespan of *Caenorhabditis elegans* (*C*. *elegans*) [20]. Additionally, we analyzed the mechanisms of the prolongevity effects on *C*. *elegans* and revealed that LG2055 feeding extended the lifespan of *C*. *elegans* by enhancing the activity of the *C*. *elegans* Nrf2 ortholog, SKN-1, concomitantly increasing the expression of its target genes and thus increasing the resistance to oxidative stress. These results indicated the anti-oxidative effects of LG2055 and suggested that LG2055 might serve as a useful antioxidant.

For considering the application of the anti-oxidative effects of LG2055, it is necessary to investigate the anti-oxidative stress effects of LG2055 in mammalian cells. Thus, in the current study, we assessed whether LG2055 was effective in protecting cells against oxidative stress and analyzed the functional molecular mechanisms.

## Materials and methods

### Preparation of lactic acid bacteria (LAB)

*Lactobacillus gasseri* SBT2055 (LG2055) was deposited in the NITE Patent Microorganisms Depositary (Chiba, Japan). *Lactobacillus gasseri* JCM1131^T^ (equal to ATCC 33323, type strain) (LG1131^T^) was purchased from Japan Collection of Microorganisms (Ibaraki, Japan). LG2055 or LG1131^T^ was cultivated at 37°C in MRS broth (Difco, Detroit, MI) for 18 h and harvested by centrifugation at 1,500 x g for 10 min at 4°C. The harvested cells were washed twice with sterile distilled water, resuspended in distilled water, and lyophilized to generate powder. LAB powder was resuspended in phosphate buffered saline (PBS) and heated at 80°C for 30 min.

### Cell culture

Mouse embryonic fibroblast (MEF) cells and NIH-3T3 cells were cultured in Dulbecco’s modified Eagle medium (high glucose) (Wako, Osaka, Japan) supplemented with 10% fetal bovine serum and incubated at 37°C in 5% CO_2_. MEF cells were isolated from embryonic day 10.5–12.5 embryos of C57BL/6N mice and cultured according to the 3T3 protocol as described in reference [21]. MEF cells at passage numbers 5–8 were used for the experiments. All animal experiments were performed in accordance with the guidelines of the Bioscience Committee of Hokkaido University and were approved by the Animal Care and Use Committee of Hokkaido University.

### Cell proliferation assay

NIH-3T3 or MEF cells were seeded in 96-well plates (5 × 10^3^ cells/well for NIH-3T3 cells or 1 × 10^4^ cells/well for MEF cells) and incubated at 37°C in 5% CO_2_. The next day, cells were treated with paraquat (0.5 mM) (Wako) with or without lactic acid bacteria (1, 10, 100 μg/mL) for 24 h. After treatment, the cells were washed with PBS and the cell proliferation rate was measured using the Cell Counting Kit-8 (Dojindo, Kumamoto, Japan) according to the manufacturer’s instructions.

### ROS detection

NIH-3T3 or MEF cells were seeded in 12-well plates (1 × 10^5^ cells/well) and incubated at 37°C in 5% CO_2_. The next day, cells were treated with paraquat (0.5 mM) with or without lactic acid bacteria (10, 50, 100 μg/mL) for 24 h. After treatment, the cells were washed with PBS and incubated with 5 μM dihydroethidium (DHE) (Wako) for 1 h at 37°C. The cells were washed again with PBS and collected by trypsinization. The fluorescence was measured using a FACSCanto II flow cytometer (BD Biosciences, Bedford, MA) and the mean fluorescence intensity was analyzed using FACSDiva software (BD Biosciences).

### GSH/GSSG ratio assay

NIH-3T3 cells were seeded in 96-well plates (5 × 10^3^ cells/well) and incubated at 37°C in 5% CO_2_. The next day, cells were treated with paraquat (0.5 mM) with or without LG2055 (100 μg/mL) for 24 h. After treatment, the cells were washed with PBS and the GSH/GSSG ratios were measured by using GSH/GSSG-Glo^™^ Assay (Promega, Madison, WI).

### RNA extraction and quantitative real time-PCR analysis

MEF cells were seeded in 24-well plates (5 × 10^4^ cells/well) and incubated at 37°C in 5% CO_2_. The next day, cells were treated with LG2055 (100 μg/mL) for 24 h. After treatment, the cells were washed with PBS and total RNA was extracted using TRIzol reagent (Invitrogen, Carlsbad, CA). First-strand cDNA synthesis was performed using the ReverTra Ace qPCR RT Master Mix with gDNA Remover (Toyobo, Osaka, Japan) according to the manufacturer’s instructions. Real time-PCR analysis was carried out using the KAPA SYBR Fast qPCR Kit (Kapa Biosystems, Wilmington, MA). The relative expression of the tested mRNA was calculated by the Delta-Delta Ct (ΔΔCt) method and normalized to *Hprt* mRNA (endogenous control). The primer sequences are listed in S1 Table.

### Western blotting

MEF cells were seeded in 6-well plates (2.5 × 10^5^ cells/well) and incubated at 37°C in 5% CO_2_. The next day, cells were treated with LG2055 (100 μg/mL) for 24 h. After treatment, the cells were washed with PBS and lysed in RIPA buffer (50 mM Tris pH 7.4, 150 mM NaCl, 1% NP-40, 0.5% deoxycholic acid, and 0.1% sodium dodecyl sulfate (SDS)) supplemented with cOmplete Mini protease inhibitor cocktail and PhosSTOP phosphatase inhibitor cocktail (Roche, Roswell, GA) and incubated at 4°C for 1 h. The residues in the cell lysates were removed by centrifugation at 3,000 x g for 5 min at 4°C. The cell lysates were separated by SDS-polyacrylamide gel electrophoresis (PAGE) and transferred to Immobilon-P membranes (Millipore, Billerica, MA). After transfer, the membranes were blocked with 5% bovine serum albumin or 5% skimmed milk in Tris-buffered saline containing 0.1% Tween 20 (TBS-T) for 1 h at room temperature and incubated overnight at 4°C with primary antibody as follows: Nrf2 (D1Z9C), Phospho-JNK Thr183/Tyr185 (81E11), JNK, Phospho-c-Jun Ser73 (D47G9), c-Jun (60A8), SOD2 (D3X8F) (all from Cell Signaling Technology, Danvers, MA), Lamin B1 (EPR8985(B), Abcam, Cambridge, UK), Hsp90 (68, BD Biosciences), and β-actin (13E5, Cell Signaling). Membranes were washed with TBS-T and incubated for 1 h at room temperature with horseradish peroxidase (HRP)-conjugated secondary antibody: anti-Rabbit IgG or anti-mouse IgG (eBioscience, San Diego, CA). HRP signals were visualized using the Immobilon Western chemiluminescent HRP substrate (Millipore) and an LAS-1000 mini image analyzer (Fujifilm, Tokyo, Japan). Band intensities were quantified by ImageJ software (National Institutes of Health, Bethesda, MD).

### Nuclear isolation

MEF cells were seeded in 100-mm dishes (8 × 10^5^ cells/dish) and incubated at 37°C in 5% CO_2_. The next day, cells were treated with LG2055 (100 μg/mL) for 24 h. After treatment, the cells were washed with PBS, collected by trypsinization, and fractionated into cytosolic and nuclear fractions using the Focus SubCell kit (G Biosciences, St. Louis, MO). The isolated fractions were analyzed by SDS-PAGE and western blotting.

### Statistical analysis

Data were expressed as the means ± standard deviations. The level of significance was determined using a one-way ANOVA and Tukey-Kramer post-test or one-way ANOVA and Dunnett’s post-test for multiple comparisons, and a Student’s *t*-test for single comparisons. A *p* value of <0.05 was considered to be statistically significant.

## Results

### LG2055 strengthens the resistance of murine cells to oxidative stress

**Fig 1:**
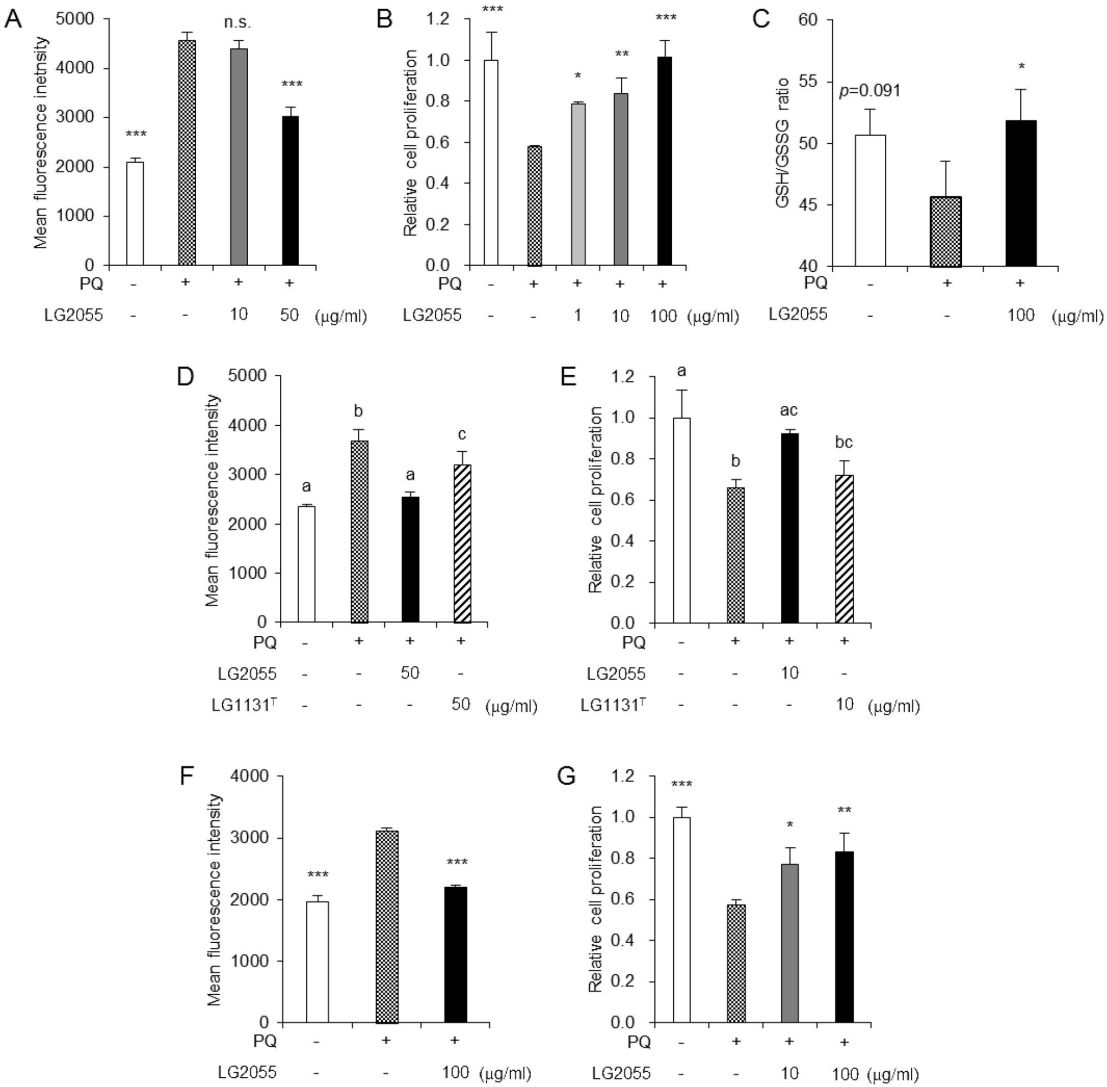
LG2055 strengthens the resistance of murine cells to oxidative stress. (A-E) NIH-3T3 cells were treated with 0.5 mM paraquat (PQ) with or without lactic acid bacteria (LG2055 or LG1131^T^) for 24 h. After incubation, (A, D) the ROS levels were measured as fluorescence intensity by DHE staining, (B, E) the cell proliferation rates were determined using a Cell Counting Kit-8 and (C) the GSH/GSSG ratios were measured by using GSH/GSSG-Glo^™^ Assay. (F, G) MEF cells were treated with 0.5 mM PQ with or without LG2055 for 24 h. After incubation, (F) the ROS levels were measured and (G) the cell proliferation rates were determined as described in Fig 1A, B. Each experiment was performed in triplicate; the data are shown as the means ± SD. (A, B, C, F, G) *p <0.05, **p <0.01, ***p <0.001 compared with the control treated with PQ alone using one-way ANOVA and Dunnett’s post-test analysis. (D, E) Values not sharing a common letter are significantly different (*p* <0.05) according to one-way ANOVA and Tukey-Kramer post-test analysis.

First, we investigated whether LG2055 regulated the oxidative stress response of murine cells to elucidate the effects of LG2055 in the mammalian cells. Mouse fibroblast cells were selected as a general model for the evaluation of oxidative stress response. These cells were widely used for the evaluation of the defense system against oxidative stress [22]. We exposed the cells to oxidative stress generated by paraquat, which is widely used as a generator of intracellular reactive oxygen species (ROS) [23]. Mouse embryonic fibroblast NIH-3T3 cells, used as an immortalized cell model, were treated with paraquat with or without LG2055 for 24 h. After the treatment, the cells were harvested and ROS accumulation levels were evaluated by dihydroethidium (DHE) staining. This analysis showed that paraquat treatment increased the ROS accumulation in NIH-3T3 cells and that LG2055 treatment suppressed the accumulation induced by paraquat (Fig 1A). Next, NIH-3T3 cells were treated with paraquat with or without LG2055 for 24 h and the cell proliferation rates were measured using a Cell Counting Kit-8. In NIH-3T3 cells, paraquat treatment induced a low rate of cell proliferation; subsequent LG2055 treatment recovered the decreased proliferation rate following paraquat treatment in a dose-dependent manner (Fig 1B). Moreover, LG2055 treatment increased the GSH/GSSG ratio, used as an oxidative stress marker, in paraquat-treated NIH-3T3 cells (Fig 1C). These results suggested that LG2055 upregulated an inherent resistance to oxidative stress in NIH-3T3 cells. Additionally, *L*. *gasseri* JCM1131^T^ (LG1131^T^), the type strain of *L*. *gasseri*, was tested in the same assays for comparison with LG2055. LG1131^T^ treatment suppressed the ROS accumulation caused by paraquat; however, the resultant ROS level was higher than that achieved in LG2055-treated cells (Fig 1D). Furthermore, LG1131^T^ treatment did not increase the proliferation rate of paraquat-treated cells (Fig 1E). From these results, we ascertained that the anti-oxidative effects of LG2055 were higher than those of LG1131^T^. The anti-oxidative effects of LG2055 were also examined in primary mouse embryonic fibroblast (MEF) cells. It is known that prolonged subculture induces the inhibition of cell proliferation (cellular senescence) in MEF cells, and increased oxidative stress is the key factor of this phenomenon [11]. Thus, MEF cells were considered to be a suitable model for studying the response against oxidative stress. Similarly, in MEF cells treated with paraquat, the ROS level was increased and proliferation rate was decreased whereas LG2055 treatment recovered these changes (Fig 1F and 1G). Taken together, these results demonstrated that LG2055 strengthened an inherent ability of resistance to oxidative stress not only in *C*. *elegans* but also in mouse cells.

### LG2055 activates the Nrf2-ARE signaling pathway

Next, we investigated the mechanisms of the anti-oxidative effects induced by LG2055. As is well known, the Nrf2-ARE signaling pathway is one of the most important defense mechanisms against oxidative stress. Our previous report revealed that LG2055 enhanced the activity of SKN-1, which is the *C*. *elegans* Nrf2 ortholog, and suppressed the decrease of anti-oxidative activity and the accumulation of oxidative damage associating with aging in *C*. *elegans* [20]. Thus, we focused on the Nrf2-ARE signaling pathway and assessed the effects of LG2055 on the Nrf2-ARE signaling pathway.

**Fig 2:**
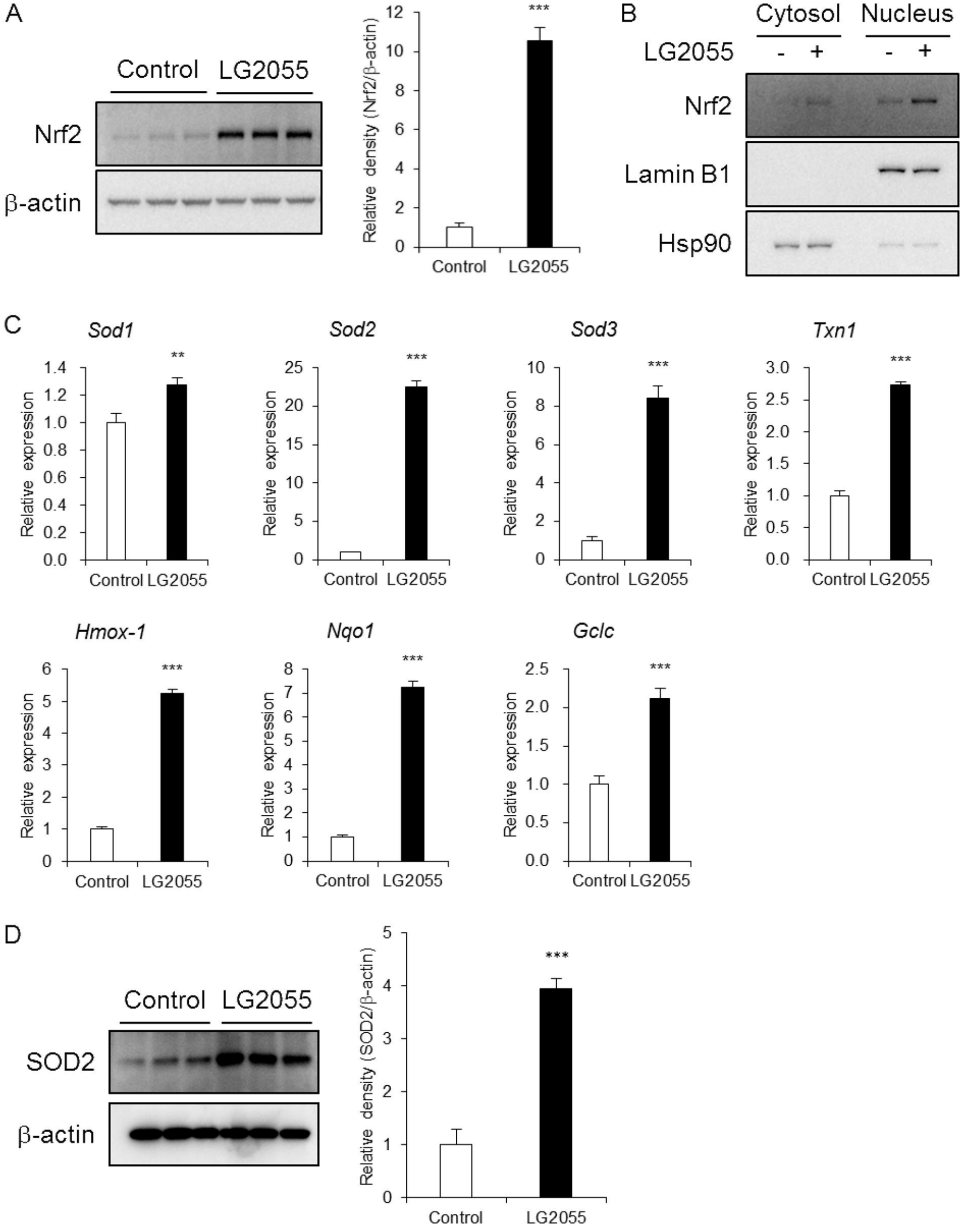
LG2055 activates the Nrf2-ARE signaling pathway. (A-D) MEF cells were cultured with or without LG2055 for 24 h. (A, D) The total cell lysates were analyzed by western blotting to compare the Nrf2 or SOD2 protein levels. The relative expression level of Nrf2 or SOD2 normalized by β-actin protein expression was quantitated. (B) The cells were fractionated into cytosolic and nuclear fractions using the Focus SubCell kit. Each fraction was analyzed by western blotting to compare the Nrf2 protein levels. Lamin B1 and Hsp90 were detected as loading controls. (C) The mRNA expression levels of cytoprotective genes were determined by quantitative real time-PCR analysis. (A, C, D) Each experiment was performed in triplicate; the data are shown as the means ± SD. **p <0.01 and ***p <0.001 according to the Student’s *t*-test.

As feeding with LG2055 upregulated SKN-1 expression in *C*. *elegans* [20], we therefore evaluated the change of Nrf2 protein levels in LG2055-treated cells. MEF cells were treated with LG2055 for 24 h and Nrf2 protein levels were analyzed by western blotting. The total Nrf2 protein levels were significantly increased in LG2055-treated cells (Fig 2A), which suggested that LG2055 might activate the Nrf2-ARE signaling pathway. In order to bind to the ARE and regulate the expression of target genes, it is necessary for Nrf2 to translocate into the nucleus. We therefore evaluated the Nrf2 protein level specifically in nuclear fractions. Cytosolic and nuclear fractions were prepared from MEF cells treated with LG2055 for 24 h and the Nrf2 protein level of each fraction was measured. The results showed that the nuclear Nrf2 protein level was increased in LG2055-treated cells (Fig 2B).

We subsequently examined whether LG2055 treatment affected the mRNA expression levels of the anti-oxidative response genes that participate in the cellular defense system against oxidative stress, including Nrf2 target genes. MEF cells were treated with LG2055 for 24 h and the mRNA expression levels of target genes were analyzed by quantitative real time-PCR analysis. LG2055 treatment upregulated the mRNA expression levels of cytoprotective genes such as *Sod1*, *2*, and *3*, thioredoxin 1 (*Txn1*), HO-1 (*Hmox1*), NAD(P)H:quinone oxidoreductase 1 (*Nqo1*), and glutamate-cysteine ligase catalytic subunit (*Gclc*) (Fig 2C). Consistent with the increase of Nrf2 protein level, this upregulation likely occurred through binding of the transcription factor Nrf2 to an ARE that exists in the promoter regions of these cytoprotective target genes to activate the Nrf2-ARE signaling pathway [8, 24]. Moreover, the protein level of anti-oxidative enzyme was increased in LG2055-treated cells (Fig 2D). Therefore, it was considered that the resistance to oxidative stress was enhanced in LG2055-treated cells.

These data indicated that LG2055 activated the Nrf2-ARE signaling pathway and upregulated the expression of Nrf2 target cytoprotective genes, resulting in the strengthened resistance against oxidative stress.

### LG2055 activates mitogen-activated protein kinase (MAPK) signaling pathway

**Fig 3:**
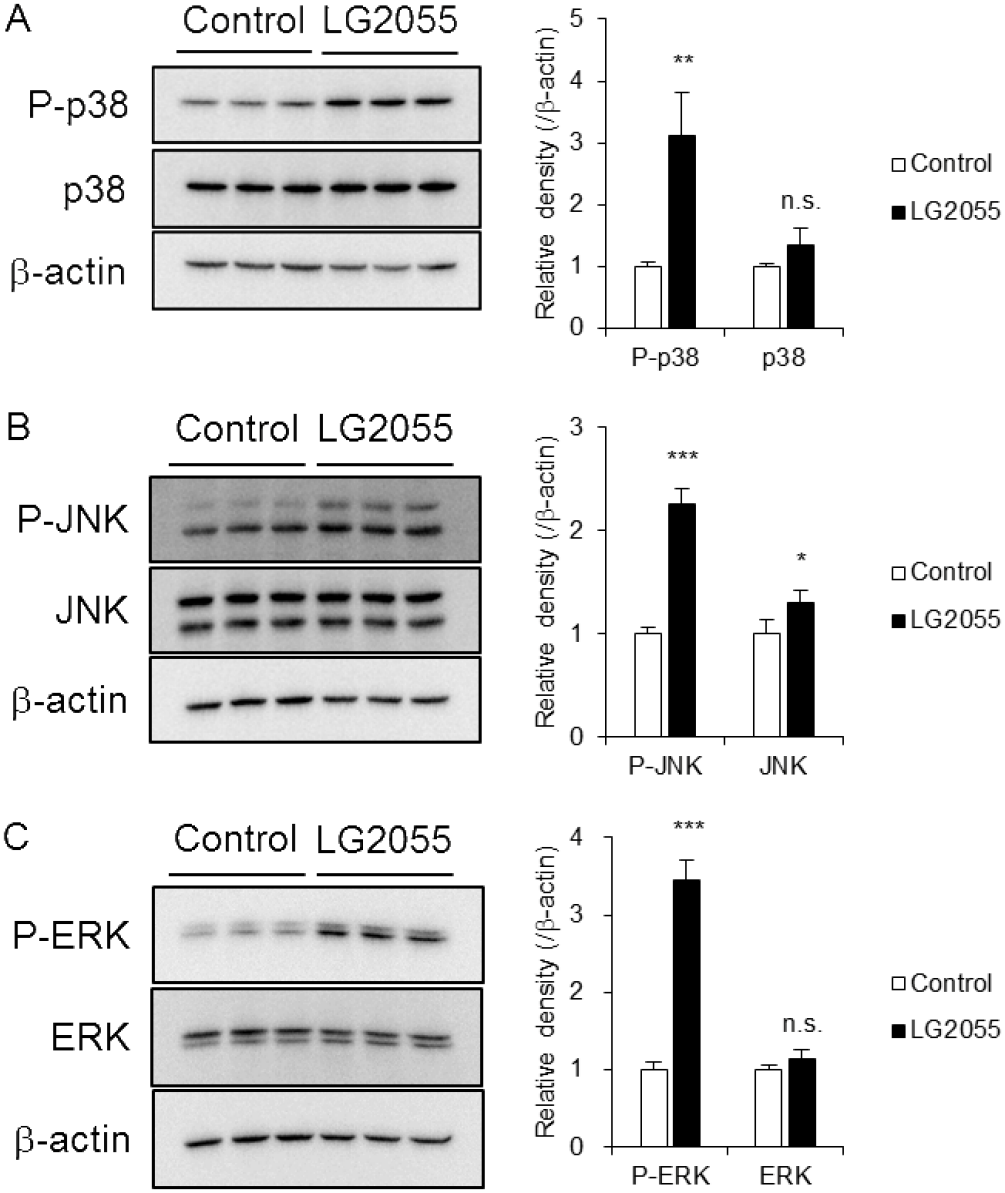
LG2055 activates MAPK signaling pathway. MEF cells were cultured with or without LG2055 for 24 h. The total cell lysates were analyzed by western blotting to compare the phosphorylated and total protein levels of p38 MAPK (A), JNK (B), and ERK (C). The relative expression level of each protein was quantitated and normalized by β-actin expression. Each experiment was performed in triplicate; the data are shown as the means ± SD. *p <0.05 and ***p <0.001 were determined by the Student’s *t*-test.

Our recent study showed that LG2055 increased the expression of SKN-1 via p38 MAPK in *C*. *elegans* [20]. MAPK is one of the kinases that regulate the Nrf2-ARE signaling pathway [25], and it was estimated that LG2055 activated the Nrf2-ARE signaling pathway through MAPK signaling in MEF cells as with in *C*. *elegans*. Considering the difference between mammals and *C*. *elegans*, we evaluated whether LG2055 activated MAPKs, including ERK, JNK and p38 MAPK in MEF cells. Our results showed that the phosphorylation levels of all three MAPKs were upregulated in LG2055-treated cells (Fig 3). It indicated that LG2055 treatment activated all three MAPKs in MEF cells, and these kinases were possible to activate the Nrf2-ARE signaling. Therefore, we subsequently performed the MAPK inhibition assays to reveal the relation between MAPKs and Nrf2 activation by LG2055.

### LG2055 activates the Nrf2-ARE signaling pathway by the regulation of JNK

**Fig 4:**
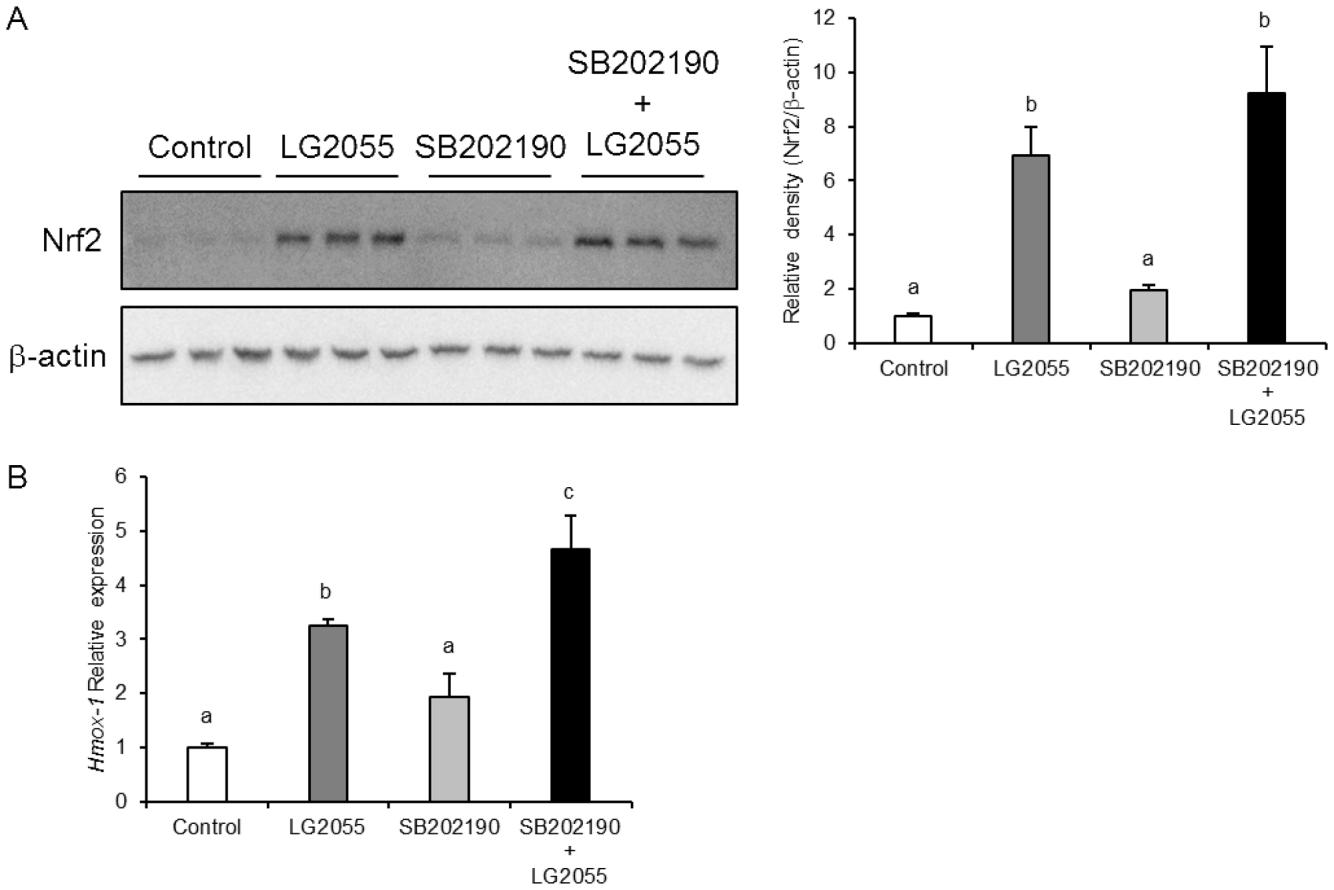
Activation of the Nrf2-ARE signaling pathway induced by LG2055 treatment is not suppressed by inhibition of p38 MAPK. MEF cells were preincubated with 10 μM SB202190 for 2 h and treated with LG2055 for 24 h. (A) The total cell lysates were analyzed by western blotting to compare the Nrf2 protein levels. The relative expression level of Nrf2 normalized by β-actin protein expression was quantitated. (B) The mRNA expression level of HO-1 was determined by quantitative real time-PCR analysis. (A, B) Each experiment was performed in triplicate; the data are shown as the means ± SD. Values not sharing a common letter are significantly different according to one-way ANOVA and Tukey-Kramer post-test analysis; *p* <0.05.

**Fig 5:**
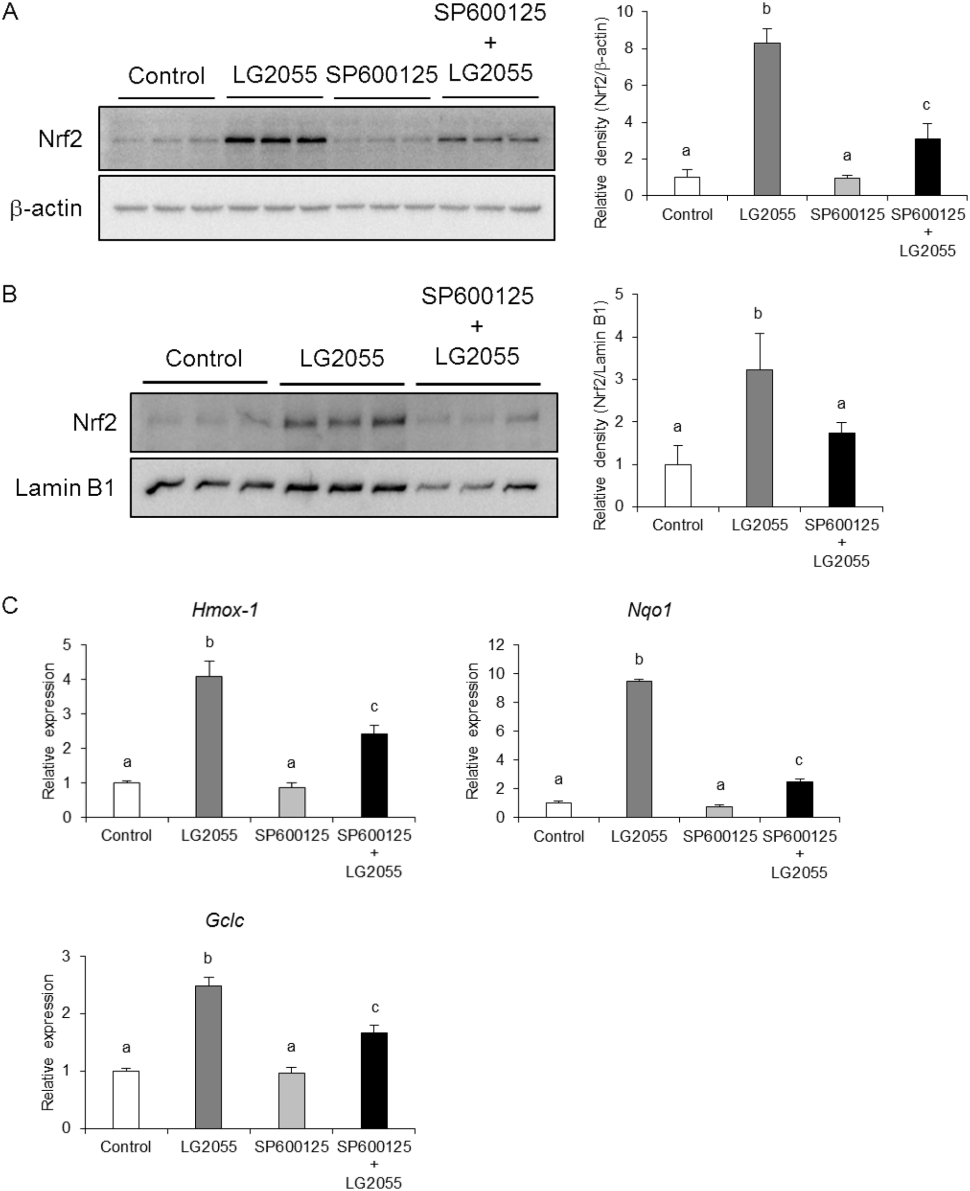
LG2055 activates the Nrf2-ARE signaling pathway by activating the JNK signaling pathway. MEF cells were preincubated with 20 μM SP600125 for 1 h and treated with LG2055 for 24 h. (A) The total cell lysates were analyzed by western blotting to compare the Nrf2 protein levels. The relative expression level of Nrf2 normalized by β-actin protein expression was quantitated. (B) The cells were fractionated into cytosolic and nuclear fractions using the Focus SubCell kit. The nuclear fraction was analyzed by western blotting to compare Nrf2 protein levels. The relative expression level of Nrf2 normalized by Lamin B1 protein expression was quantitated. (C) The mRNA expression levels of Nrf2 target genes were determined by quantitative real time-PCR analysis. (A-C) Each experiment was performed in triplicate; the data are shown as the means ± SD. Values not sharing a common letter are significantly different according to one-way ANOVA and Tukey-Kramer post-test analysis; *p* <0.05.

**Fig 6:**
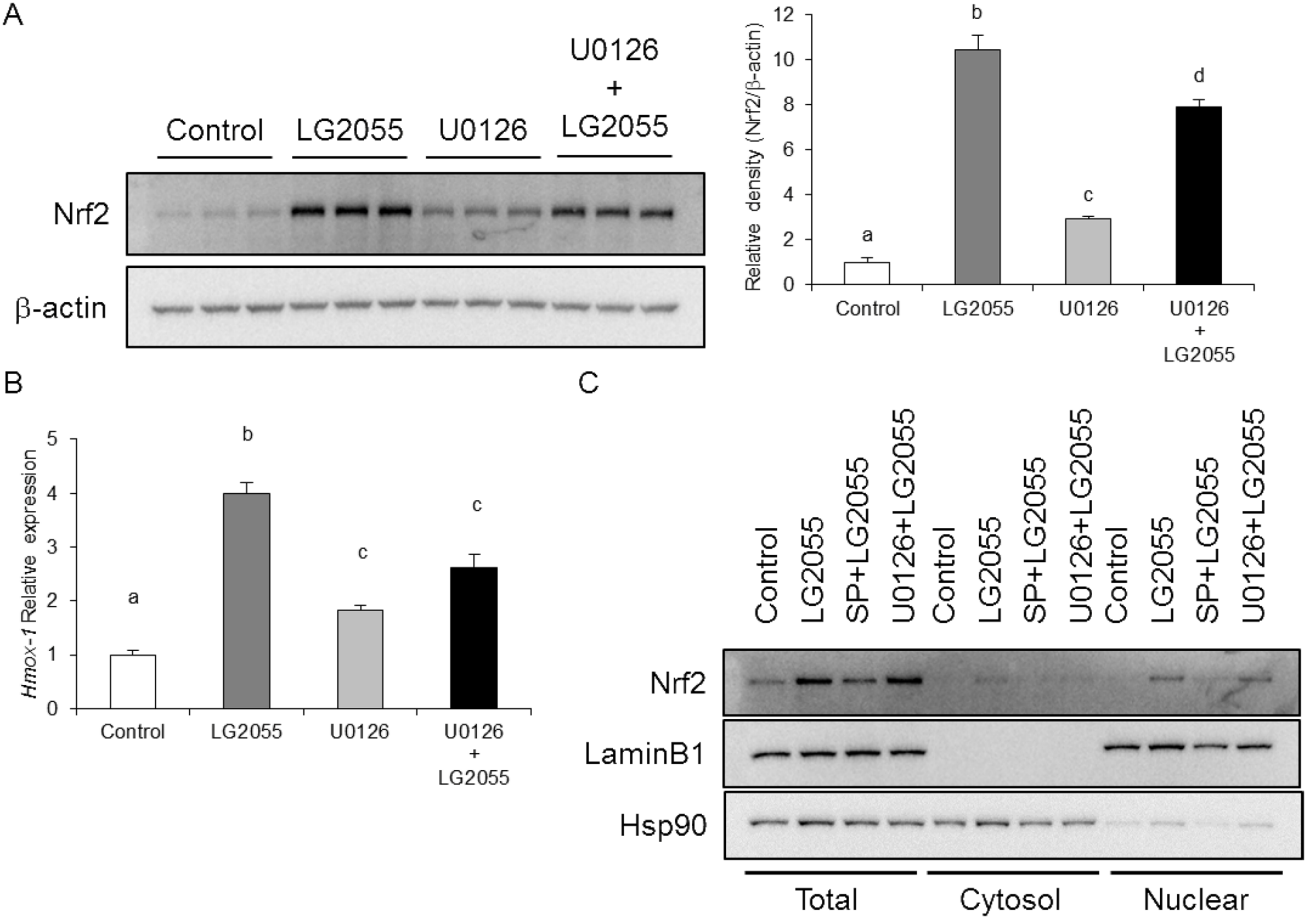
Activation of the Nrf2-ARE signaling pathway induced by LG2055 treatment is slightly suppressed by inhibition of ERK. (A, B) MEF cells were preincubated with 20 μM U0126 for 1 h and treated with LG2055 for 24 h. (A) The total cell lysates were analyzed by western blotting to compare the Nrf2 protein levels. The relative expression level of Nrf2 normalized by β-actin protein expression was quantitated. (B) The mRNA expression level of HO-1 was determined by quantitative real time-PCR analysis. (C) MEF cells were preincubated with 20 μM SP600125 (SP) or 20 μM U0126 for 1 h and treated with LG2055 for 24 h. The cells were fractionated into cytosolic and nuclear fractions using the Focus SubCell kit. Each fraction was analyzed by western blotting to compare Nrf2 protein levels. (A, B) Each experiment was performed in triplicate; the data are shown as the means ± SD. Values not sharing a common letter are significantly different according to one-way ANOVA and Tukey-Kramer post-test analysis with the value of *p* <0.05.

To further address the role of individual MAPK pathways in activation of the Nrf2-ARE signaling pathway induced by LG2055, specific kinase inhibitors were utilized. MEF cells, preincubated with the each inhibitor, were treated with LG2055 for 24 h and the total cell Nrf2 protein levels were determined. At first, it was shown that p38 MAPK inhibitor SB202190 did not suppress the increase of Nrf2 protein level (Fig 4A) and the mRNA expression of HO-1 under LG2055 treatment (Fig 4B). In contrast, the JNK inhibitor SP600125 significantly suppressed the increase of Nrf2 protein levels under LG2055 treatment (Fig 5A). We checked the phosphorylation level of c-Jun, which is a target of JNK, and SP600125 completely suppressed the increase of c-Jun phosphorylation level induced by LG2055, indicating that SP600125 pretreatment successfully inhibited the activation of JNK signaling (S1 Fig). Furthermore, the inhibition of JNK also suppressed the increase in the level of nuclear Nrf2 protein (Fig 5B), which is essential for activation of the Nrf2-ARE signaling pathway. Additionally, the mRNA expression levels of Nrf2 target genes were determined to evaluate the significance of JNK inhibition on the Nrf2-ARE signaling pathway. Pretreatment with the JNK inhibitor suppressed the LG2055-induced increased expression levels of the Nrf2 target genes *Hmox1*, *Nqo1*, and *Gclc* (Fig 5C). These data suggested that the activation of JNK was required for the increase of Nrf2 protein level and for the activation of the Nrf2-ARE signaling pathway induced by LG2055. In addition, U0126, which suppresses the activation of ERK signaling by inhibiting MEK1/2, slightly suppressed the increase of Nrf2 protein level (Fig 6A) and the mRNA expression of HO-1 induced by LG2055 (Fig 6B), and did not suppress the increase of nuclear Nrf2 level in LG2055-treated cells (Fig 6C). Therefore, it was suggested that the contribution of ERK to the induction of Nrf2 expression by LG2055 might be small compared to that of JNK.

## Discussion

Recently, many studies have been undertaken regarding the availability and functionality of lactic acid bacteria. The beneficial effects of lactic acid bacteria are achieved by various mechanisms. For example, the bacterial products or the components of bacterial bodies can act as biologically active substances. In this case, it is not necessary that living bacteria exist in the host [26, 27]. It has been reported that heat-killed *Lactobacillus rhamnosus* GG (LGG) showed anti-inflammatory effects as well as living LGG, and some bacterial components of heat-killed LGG have been shown to be effective [28]. Alternatively, probiotics, which are defined as ‘living microorganisms that when administered in adequate amounts confer health benefits on the host’, show their effects in the host as living bacteria.

The anti-oxidative effects of these bacteria have also been reported. For example, it was reported that *Lactobacillus acidophilus* 606 exhibited anti-oxidative effects *in vitro* [15] and that feeding with *Lactobacillus plantarum* FC225 could elevate the activities of SOD and glutathione peroxidase and decrease the content of malondialdehyde, a marker of oxidative stress, in mice fed a high fat diet [29]. As increased oxidative stress has been shown to cause various diseases [1–6], controlling this factor is therefore important for disease prevention. Lactic acid bacteria, which are considered to be safe food ingredients, can be used for this purpose. As we had previously demonstrated that dietary supplementation with LG2055 strengthened the resistance against oxidative stress and enhanced prolongevity in *C*. *elegans* [20], we expected that LG2055 might also exhibit beneficial effects in mammals. Thus, in the current study, we investigated the anti-oxidative stress effects of LG2055 on mammalian cells and analyzed the functional mechanisms of these effects.

At first, we showed that LG2055 treatment suppressed the increase of ROS levels and the decrease of cell proliferation rates caused by paraquat-induced oxidative stress in NIH-3T3 cells and MEF cells (Fig 1). These data suggested that LG2055 showed the anti-oxidative effects in mouse cells as well as in *C*. *elegans*. Therefore, we subsequently investigated whether LG2055 activated the Nrf2-ARE signaling pathway, one of the most important anti-oxidative defense mechanisms. We assessed the Nrf2 protein level in LG2055-treated MEF cells. Our results demonstrated that the total Nrf2 protein level was notably increased in LG2055-treated MEF cells (Fig 2A). Additionally, the increase of Nrf2 was significant in the nuclear compared with the cytosolic fraction (Fig 2B). Nrf2 nuclear translocation is important for the activation of the Nrf2-ARE signaling pathway, since binding of the transcription factor Nrf2 to the ARE in the promoter region of its target cytoprotective genes is essential for the expression of these genes. We therefore evaluated the mRNA expression levels of anti-oxidative and cytoprotective genes in LG2055-treated cells, and the mRNA expression levels of *Sod1-3*, *Txn1*, *Hmox1*, *Nqo1*, and *Gclc* were increased (Fig 2C). These data suggested that LG2055 effectively activated the Nrf2-ARE signaling pathway and induced the expression of these genes to strengthen the defense system against oxidative stress.

We showed that LG2055 induced the expression and nuclear translocation of Nrf2 to generate anti-oxidative effects via activation of the Nrf2-ARE signaling pathway. It has been reported that some other lactic acid bacteria activate the Nrf2-ARE signaling pathway. For example, it has been shown that *L*. *plantarum* FC225 increased Nrf2 expression, thus protecting liver cells against oxidative injury induced by a high fat diet in mouse [29] and that some lactobacilli activated the Nrf2-ARE signaling pathway via NADPH oxidase 1, which was essential for the associated cytoprotective effect [30]. Notably, the Nrf2-ARE signaling pathway is also implicated in the protection against neurodegenerative [8, 31], hepatic [32], gastrointestinal [32], and cardiovascular diseases [33, 34]. Each study was designed to use the optimal cells or tissues in these references, and suggested that the Nrf2-ARE signaling pathway was a promising therapeutic target for these diseases. In this study, we revealed that LG2055 activated the Nrf2-ARE signaling pathway in mammalian cells, using mouse fibroblast cells as a general model. LG2055, the activator of Nrf2, was expected to have a potential for prevention of these diseases, and more studies, using the suitable cells or tissues for the target disease, are required to reveal the effects of LG2055.

Next, we assessed the mechanisms of the activation of the Nrf2-ARE signaling pathway induced by LG2055 treatment. The activation of the Nrf2-ARE signaling pathway is reported to be induced by several signaling molecules [25]. Kelch-like ECH-associated protein 1 (Keap1) is the repressor protein of Nrf2 and remains Nrf2 inactive under non-stressed conditions. Another known mechanism involves the regulation of the Nrf2-ARE signaling pathway by several kinases. In particular, protein kinase C (PKC) promotes the activation of the Nrf2-ARE signaling pathway by Nrf2 phosphorylation. The phosphatidylinositol 3 kinase (PI3K)/protein kinase B (Akt) pathway also participates in the activation of the Nrf2-ARE signaling pathway. Furthermore, it has also been reported that the MAPK pathway, including ERK, JNK, and p38 MAPK, activates the Nrf2-ARE signaling pathway as well [25].

Our previous study revealed that LG2055 activated SKN-1, the *C*. *elegans* Nrf2 ortholog, and showed the anti-oxidative effects through the p38 MAPK signaling pathway (NSY-1-SEK-1-PMK-1-SKN-1) in *C*. *elegans* [20]. As described above, MAPK signaling is one of the mechanisms that regulate the Nrf2-ARE signaling pathway. Depending on our results in *C*. *elegans* [20], we hypothesized that LG2055 activated the Nrf2-ARE signaling pathway through MAPK signaling. Considering the difference between *C*. *elegans* and mammals, we assessed whether MAPK signaling, including p38 MAPK, JNK, and ERK, contributed to Nrf2 activation in MEF cells. We found that whereas all three MAPKs were activated in LG2055-treated MEF cells, the inhibition of each MAPK showed different influence on the Nrf2-ARE signaling pathway.

The inhibition of p38 MAPK did not suppress the increase of Nrf2 protein level and the expression of the Nrf2 target genes induced by LG2055 treatment (Fig 4). From these data, we considered that the contribution of p38 MAPK for Nrf2 activation is not high in LG2055-treated cells and that Nrf2 activation induced by LG2055 was regulated by other factor(s). It has been reported that p38 MAPK activates or suppresses the Nrf2-ARE signaling pathway depending on cell type [35–38]. Taken together, these findings suggested that the mechanisms of Nrf2 and SKN-1 activation caused by LG2055 might differ between in mammals and *C*. *elegans*.

MEK1/2 inhibitor U0126 suppresses the phosphorylation of ERK, and U0126 treatment slightly suppressed the increase of Nrf2 expression and Nrf2 target genes induced by LG2055. However, U0126 treatment did not inhibit the nuclear Nrf2 level in LG2055-treated cells (Fig 6). These results suggested that the contribution of ERK for Nrf2 activation by LG2055 was not large compared to that of JNK.

In fact, the inhibition of JNK by SP600125 notably suppressed the increase of total and nuclear Nrf2 protein levels and the expression of Nrf2 target genes, suggesting that the activation of the Nrf2-ARE signaling pathway by LG2055 was dependent on JNK signaling (Fig 5). It is known that JNK is phosphorylated by MKK4/7, activated by several MAPKKKs, including ASK1 [39]. We have shown in the previous report that LG2055 enhanced the expression of NSY-1, which is orthologous to the mammalian ASK family of protein kinases in *C*. *elegans* [20]. Therefore, there is a possibility that LG2055 induces the expression of ASK family kinase to activate JNK. Further studies are required for the elucidation of the detailed mechanism of JNK activation by LG2055.

We revealed that JNK inhibition significantly suppressed Nrf2 induction caused by LG2055, whereas ERK inhibition slightly suppressed it. These results indicated that the activation of the Nrf2-ARE signaling pathway was mainly dependent on JNK signaling. On the other hand, it has been reported that several kinases, such as PKC and PI3K, also regulate the Nrf2-ARE pathway [25]. We did not investigate whether these kinases have a function of Nrf2 expression induced by LG2055, and it remains to be elucidated in the future.

**Fig 7:**
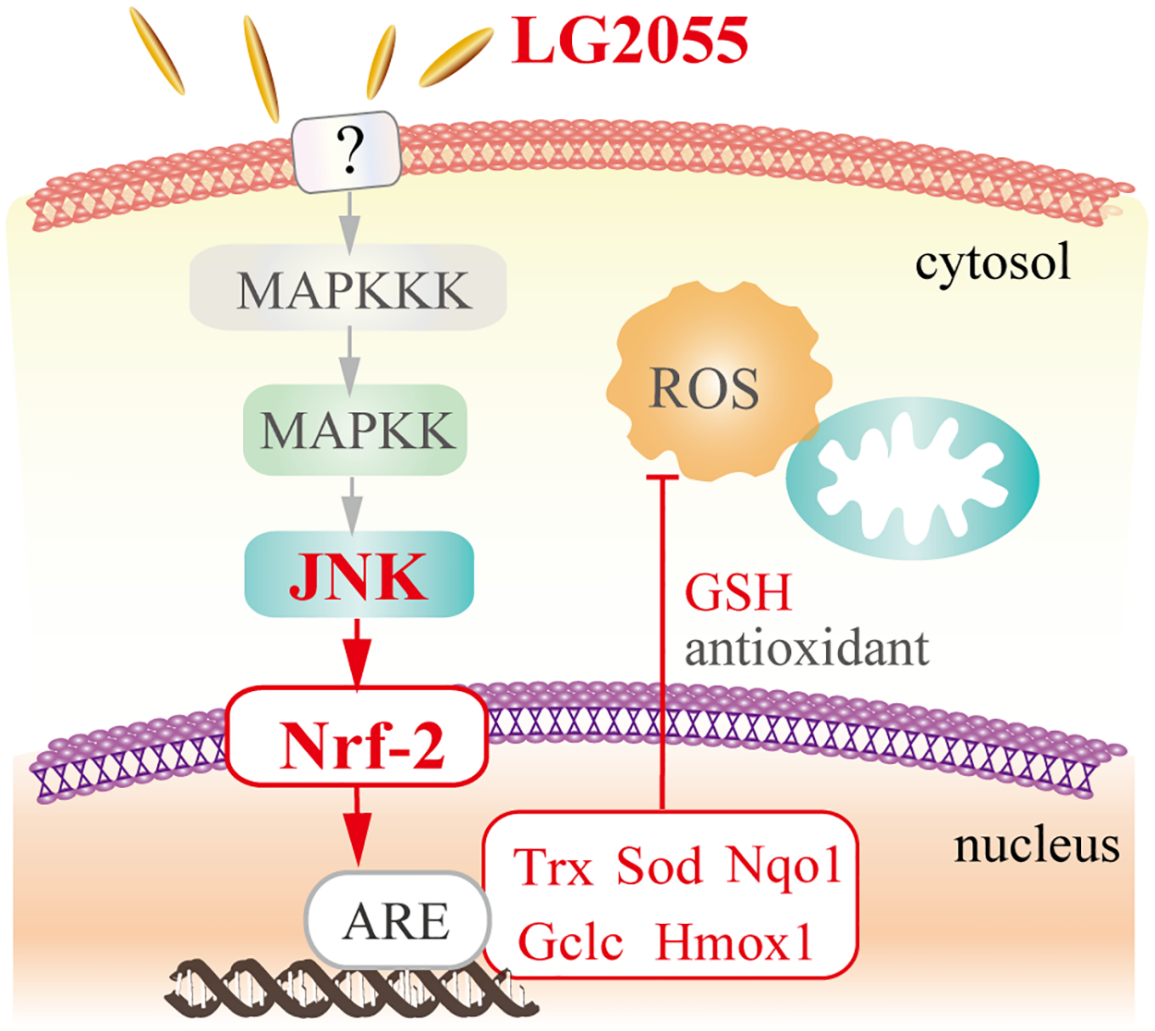
Schematic representation of the strengthened resistance against oxidative stress induced by LG2055. LG2055 induces the activation of JNK signaling and the subsequent Nrf2-ARE signaling pathway. Then induced expression of the antioxidant molecules enhances the antioxidant response.

Taken together, we demonstrated that LG2055 strengthened the resistance of mouse fibroblast cells to oxidative stress, and that the activation of Nrf2-ARE signaling pathway caused by LG2055 contributed to the anti-oxidative effects. The activation of Nrf2-ARE signaling pathway was mainly dependent on JNK activation induced by LG2055. It was suggested that LG2055 activated JNK signaling, and it contributed to the increase of total and nuclear Nrf2 protein levels. Subsequently, the transcription factor Nrf2 upregulated the mRNA expression of its target cytoprotective genes, such as *Hmox-1*, *Nqo1*, and *Gclc*, and the resistance against oxidative stress was strengthened (Fig 7).

LG2055 is one of the probiotic lactic acid bacteria; however, it was used as heat-killed bacterial bodies and showed protective effects against oxidative stress in this study. This suggested that some active substances likely contributed to the anti-oxidative effects of LG2055. Many functional effects of the substances derived from bacterial bodies have been studied and the effects of extracellular polysaccharide [40], lipoteichoic acid [26], and peptidoglycan [41] have been reported. As it is known that the structures of bacterial components differ between species or strains of the bacteria, it is expected that the differences in the functions of several lactic acid bacteria might be caused by structural differences between species or strains [42]. In this study, we demonstrated that the anti-oxidative stress effects promoted by LG2055 were higher than those of LG1131^T^ (Fig 1D and 1E). This might therefore be due to a difference in the amount or structure of biologically active substances between these strains [42, 43]. It was expected that LG2055 contained more amount of some biologically active substances than LG1131^T^, or those of LG2055 had higher activity. After all, further studies are required to identify the specific biologically active substances that provide the observed anti-oxidative stress effects mediated by LG2055.

In conclusion, our results demonstrated that LG2055 treatment strengthened the defense system against oxidative stress in mammalian cells via the JNK-dependent Nrf2-ARE signaling pathway. LG2055 might therefore serve as a basis for the development of therapeutic or protective applications for oxidative stress-related diseases.

## Supporting information

S1 Fig SP600125 pretreatment suppresses the phosphorylation of c-Jun in LG2055-treated cells. MEF cells were preincubated with 20 μM SP600125 for 1 h and treated with LG2055 for 24 h. Total cell lysates were analyzed by western blotting to compare the phosphorylated and total protein levels of c-Jun. The relative expression level of c-Jun normalized by β-actin expression was quantitated. Each experiment was performed in triplicate; the data are shown as the means ± SD. Values not sharing a common letter are significantly different according to one-way ANOVA and Tukey-Kramer post-test analysis with the value of *p* <0.05.
(TIF)

S1 Table Gene sequences of the primer sets used for quantitative real time-PCR analysis. (TIF)
