# Supplementary figures and images for: Protective effects and functional mechanisms of Lactobacillus gasseri SBT2055 against oxidative stress
Source: PLoS One. 2017 May 11;12(5):e0177106. doi: 10.1371/journal.pone.0177106 (PMC5426657; doi:10.1371/journal.pone.0177106)

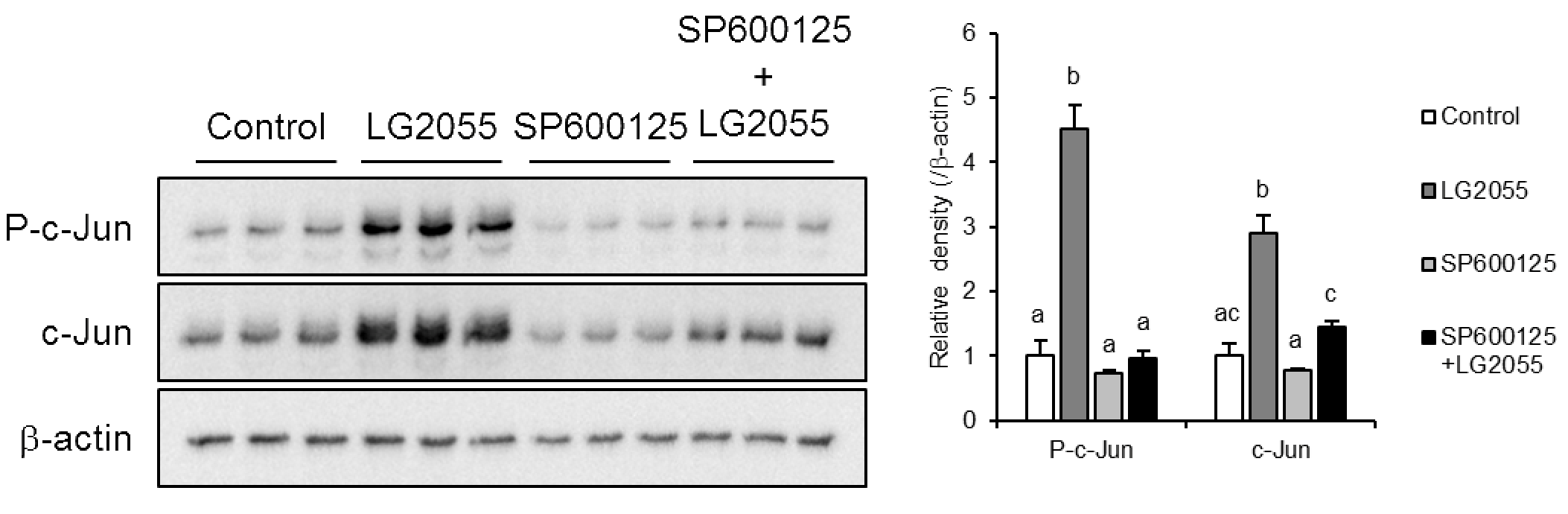

Supplement: S1 Fig — MEF cells were preincubated with 20 μM SP600125 for 1 h and treated with LG2055 for 24 h. Total cell lysates were analyzed by western blotting to compare the phosphorylated and total protein levels of c-Jun. The relative expression level of c-Jun normalized by β-actin expression was quantitated. Each experiment was performed in triplicate; the data are shown as the means ± SD. Values not sharing a common letter are significantly different according to one-way ANOVA and Tukey-Kramer post-test analysis with the value of p <0.05. (TIF) [file pone.0177106.s001.tif]

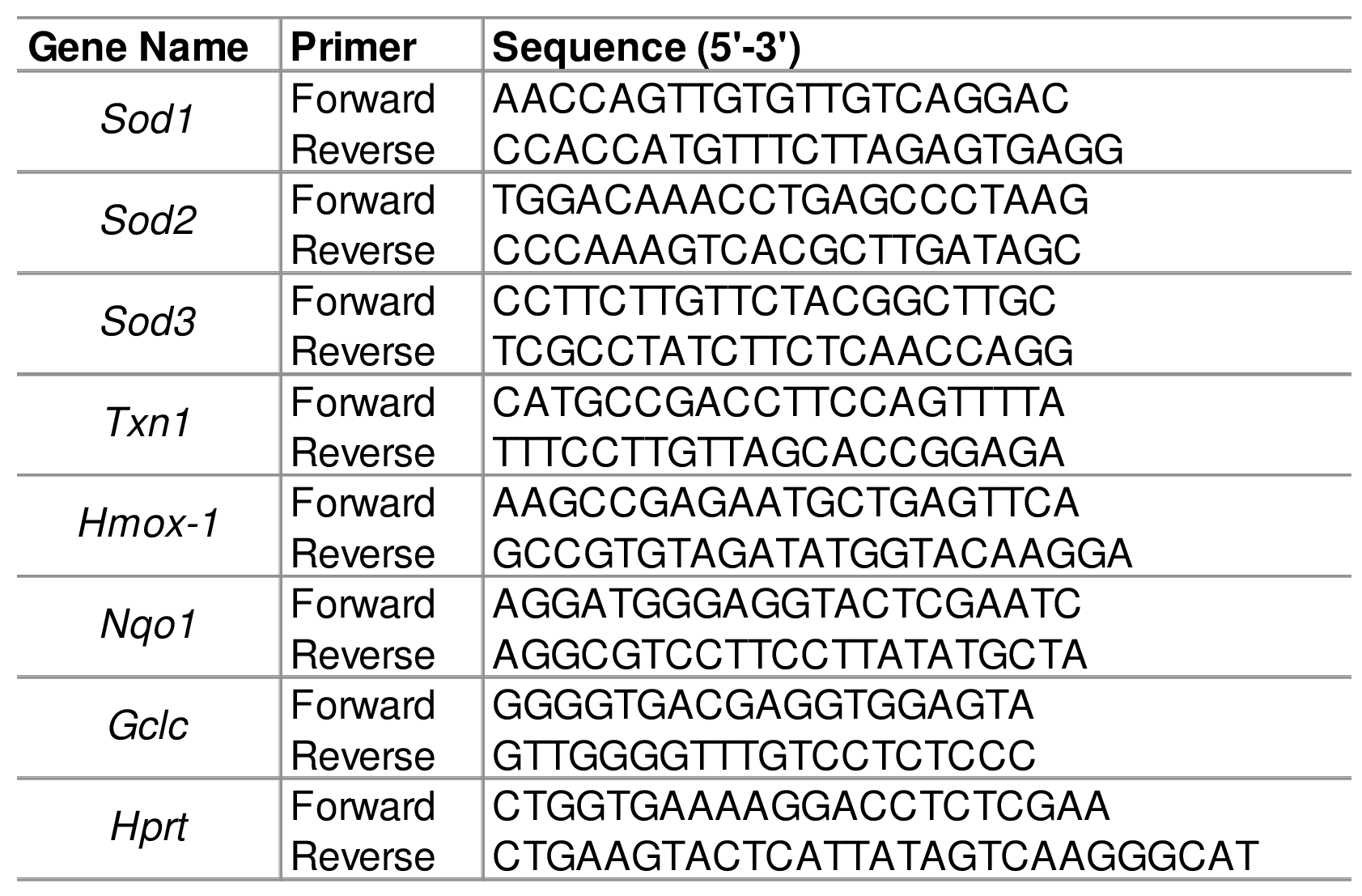

Supplement: S1 Table — (TIF) [file pone.0177106.s002.tif]
